# White Shark Offshore Habitat: A Behavioral and Environmental Characterization of the Eastern Pacific Shared Offshore Foraging Area

**DOI:** 10.1371/journal.pone.0008163

**Published:** 2009-12-09

**Authors:** Nicole Nasby-Lucas, Heidi Dewar, Chi H. Lam, Kenneth J. Goldman, Michael L. Domeier

**Affiliations:** 1 Marine Conservation Science Institute, Fallbrook, California, United States of America; 2 Southwest Fisheries Science Center, National Marine Fisheries Service, La Jolla, California, United States of America; 3 Marine Environmental Biology, University of Southern California, Los Angeles, California, United States of America; 4 Alaska Department of Fish and Game, Homer, Alaska, United States of America; NOAA/NMFS/SWFSC, United States of America

## Abstract

**Background:**

Although much is known about the behavior of white sharks in coastal regions, very little is known about their vertical movements offshore in the eastern Pacific where they spend up to five months. We provide the first detailed description of the offshore habitat use of white sharks in the eastern North Pacific.

**Methodology/Principal Findings:**

This study uses 2-min data from four recovered pop-up satellite archival tags deployed at Guadalupe Island (2002 and 2005). Deployments ranged from 5.4 to 8.2 months. Two predominant vertical patterns were described. The first was a bimodal vertical pattern with time spent at the surface and at depth, which was observed while traveling. The second was a repetitive oscillatory diving mode displayed by sharks in the Shared Offshore Foraging Area (SOFA). For all four datasets the average maximum daily dive depths ranged from 442.5 to 492.8 m and were typically associated with dissolved oxygen concentrations of above 1.7 ml L^−1^. Although infrequent, occasional dives to near 1000 m with a minimum temperature of 3.9°C and a minimum O_2_ level of 0.3 ml L^−1^ were observed.

**Conclusions/Significance:**

Recovered pop-up satellite tags from Guadalupe Island white sharks advance our understanding of the vertical habitat use of white sharks while offshore. The bimodal vertical pattern during traveling is most likely related to geolocation. The oscillatory dive pattern is likely associated with foraging. While feeding is not documented, foraging is likely occurring in association with the deep scattering layer. Diving depths were not limited by temperature but were constrained by O_2_ levels below approximately 1.5 ml L^−1^. While oxygen may limit the extent of sharks' vertical movements, it will also impact prey distribution. Consequently, the shallow oxygen minimum zone in the SOFA may act to concentrate prey, thus enhancing foraging opportunities in these oligotrophic waters.

## Introduction

Innovations in satellite tagging technology over the last 10 years have fundamentally improved our understanding of pelagic shark biology. A prime example is the ability to track offshore movements of white sharks (*Carcharodon carcharias*) reported in a number of studies. Here we take the next step and provide a comprehensive analysis of offshore vertical habitat use by white sharks. Highly detailed archival records from recovered satellite tags provide insights into vertical movements and allow for more detailed characterization of offshore habitat in relation to temperature and oxygen.

White sharks have a broad global distribution and are most commonly observed in coastal waters near marine mammal rookeries where they forage [1]–[4]. In addition to being the largest predatory sharks, they are endothermic [5], similar to other members of the family Lamnidae. White sharks can elevate muscle, stomach, brain, and eye temperatures [5]–[9] and it has even been suggested that they can defend a specific body core temperature [10], more similar to homeotherms than other endothermic fish. White sharks also possess unique features of hematology and cardiac morphology relative to other elasmobranch fishes. They have hematocrit and hemoglobin (Hb) levels that are higher than in most birds and mammals, and they have a large heart with a thick muscular ventricle [11], [12].

The majority of research on white sharks has been conducted near shore, and until recently, white sharks were thought to be primarily coastal in distribution. A number of recent studies, however, have revealed the importance of offshore movements for sub-adult and adult sharks of this species. The most comprehensive of these studies utilized satellite tags at white shark aggregations sites off the Central California coast [13], [14] and Guadalupe Island, Mexico [15]. Tracks of white shark movements obtained from both areas over multiple years reveal a consistent pattern of seasonal migration from the nearshore feeding grounds to a common offshore region centered halfway between Baja California and the Hawaiian Islands [13]–[15], which has been termed the Shared Offshore Foraging Area (SOFA) [15]. White sharks from central California and Guadalupe Island reside in this pelagic zone for up to five months, with a small percentage of the sharks traveling as far west as Hawaii. Males return to their original aggregation site the same year, while sexually mature females typically return in alternate years [16], [17].

Long-term residency in a pelagic environment has not been described for white sharks from other regions, although large-scale movements have been reported. In the southern hemisphere, Bonfil et al. [18] documented an extensive migration of one female white shark from South Africa to Australia over approximately four months. This same shark was later re-sighted at the point of origin in South Africa. Other data for white sharks tagged in South Africa exhibited shorter coastal movements [19]. Bruce et al. [20] reported movement from Australia to New Zealand but found that most movements were confined to shelf waters along the coast of Australia.

In previous studies conducted in the eastern Pacific, some analyses of vertical habitat use by white sharks have been performed [4], [14], [15], [21]. In coastal habitats, vertical movements were typically restricted by bathymetry, and depth distributions tend to be shallow. While migrating offshore, sharks spend the majority of their time near the surface with occasional deep dives. Once offshore in the SOFA, they spend less time near the surface and more time below the mixed layer, especially during the day [14], [15].

While some information on offshore depth distributions and dive patterns for white sharks is available, a detailed analysis of vertical behavior patterns has not yet been conducted. Pop-up satellite archival tags only transmit daily summaries of temperature and depth data, limiting inferences about detailed behaviors. A few recovered tags have provided high-resolution datasets of temperature, depth, and light in this pelagic region. However, Weng et al. [14] obtained only two such long-term archival datasets, one from a female and one from a shark of unknown sex. Domeier and Nasby-Lucas [15] presented a limited treatment of the four long-term archival datasets reported in their study.

A more detailed examination of behaviors is important to further elucidate how white sharks are using the pelagic environment. Detailed analyses of vertical distribution data can provide insights into the activities within the SOFA and facilitate a greater understanding of the motivations for these large-scale seasonal migrations. Better information on how behaviors are influenced by water column structure in three dimensions relative to oceanographic variables such as temperature and oxygen will improve the characterization of essential habitat and help define potential barriers to movement. An accurate definition of white shark habitat will better enable the prediction of potential shifts in distribution associated with long or short-term climate change. Information on spatial overlap with fisheries will indicate potential sources of bycatch mortality. Finally, given that white shark populations are threatened globally [22], a better understanding of their migratory cycle and habitat preferences is critical for conservation efforts.

The objective of this paper is to use data from recovered pop-up satellite archival tags to describe the behavior and habitat use of white sharks from Guadalupe Island while in the pelagic environment. We examine archival records for males and females and present the first detailed description of the vertical behavioral patterns and characterize the pelagic habitat for these sharks relative to depth, ambient temperature and oxygen concentration. Examination of vertical movements in relation to environmental data from a range of sources allows greater understanding of the physiological capabilities and constraints that influence behavior.

## Materials and Methods

White sharks were tagged at Guadalupe Island, Mexico, which is located 407 km south–southwest of San Diego, California, and 260 km offshore of Baja California, Mexico (29.12°N, 118.27°W). The island rises out of deep water (3000 m deep ∼15 km off the island) and stretches 41 km in a north/south direction and 15 km across at the widest point. Sharks were tagged using pop-up satellite archival tags (PAT, Wildlife Computers, Redmond WA, USA). Each shark was lured alongside the research vessel and tagged using a hand-held tagging pole to insert a nylon tag head into the dorsal musculature at the base of the dorsal fin. Tags were rigged with an umbrella-style dart (described in Domeier et al. [23]) and either 136 kg test Sufix Superior monofilament (Yao I Fabric) or 113 kg test nylon coated braided stainless steel leader (Sevenstrand). PAT tag models employed included PAT2 and PAT4. Wildlife Computer tags remained attached to the fish until a pre-programmed date and time when an electrolytic release mechanism caused detachment and the tag floated to the surface and transmitted data to the Argos satellite system. Data are transmitted in a summarized form. Recovered tags, however, provide high-resolution (2-min sampling frequency) archival datasets of temperature, depth and relative light level. In this study, only archival datasets for tags that remained attached for at least 160 days were included in the analysis. All sharks were photographed underwater to determine sex and identify individuals using the Guadalupe Island white shark photo-identification database [17].

Movements were estimated with recorded light and temperature data using an extended version of the TrackIt model [24]. This model estimated two positions per day using raw time-series data independent of any tag manufacturer's light-based positional estimates. The extended version incorporated sea-surface temperature (SST) matching, using the same implementation as described in Lam et al. [25]. All SST observations were matched with the low-resolution 1-degree Reynolds optimum interpolation SST dataset (http://atlas.nmfs.hawaii.edu/cgi-bin/reynolds_extract.py). Higher-resolution imagery products provided similar estimated tracks. Given that the study area is mostly in the open ocean, the low-resolution Reynolds dataset was used to maximize computational efficiency. Using this implementation of the TrackIt model, median uncertainty in the calculated position points for longitude was 1.1–1.3° and for latitude was 1.1–1.7°, where the higher uncertainty bound can be attributed to the poor light data collected during dives to deeper depths.

Vertical behavior patterns were analyzed by performing an activity analysis. The extent of vertical movements was quantified over each hour to assess relative levels of activity during diving. Vertical activity analysis (V_activity20_) was performed by counting the number of vertical movements greater than 20 m between successive 2-min interval data points over each one-hour period. This indicated whether the sharks tended to swim at a constant depth or exhibit intense oscillatory diving patterns. Twenty meters was chosen for activity analysis over other ranges because it provided the greatest clarity between different behavioral patterns. V_activity20_ values were plotted for the deployment period of each tag and over a 48-hour period in order to display any diel patterns. In all analyses of data from day and night, daytime included the period of nautical twilight. In order to further differentiate vertical behavioral patterns, rates of ascent and descent of dives were analyzed. To eliminate minor vertical movements, dives used in this analysis were those longer than 20 minutes where the temperature changed by more than 10°C.

Analyses were performed to evaluate the association of white sharks in the SOFA with the deep scattering layer (DSL). To examine the relationship between depth and lunar illumination, percent illumination was regressed on mean nighttime depth. Due to the uneven distribution of points across illumination, the average mean depth for 10% illumination intervals were calculated for the regression analysis. Levels of illumination were obtained from the Navy Astronomical Applications Department (http://aa.usno.navy.mil/data/docs/MoonFraction.php). For the calculation of the depth of the DSL, monthly composites of diffuse attenuation coefficient at 490 nm (Kd490) from two satellites (MODIS-Aqua and SeaWifs) were processed through NASA's Giovanni remote sensing data tool (http://disc.sci.gsfc.nasa.gov/giovanni/) for the area of the SOFA.

Oxygen levels experienced at depth were approximated by using the calculated track points and matching them up with data from the World Ocean Atlas 2005 [26]. The World Ocean Atlas 2005 provides global, 1° gridded, objectively analyzed climatological fields of environmental parameters and their associated errors. Dissolved oxygen values (statistical mean and standard error of the mean) were extracted for each matching track position. Based on the extracted data, an oxygen value was assigned for each 2-min data point. It should be noted that ambient oxygen levels are being inferred from climatology data rather than being measured concurrently by the tag. Some mismatch in the spatial/temporal resolution of these datasets is to be expected, but these values provide a general picture of the actual oxygen levels experienced by the sharks. The impact of the uncertainty of the position points on the extracted oxygen values is minimized since we were interested in values at the maximum daily depths and dissolved oxygen levels are more homogeneous at deeper depths, and the contours of dissolved oxygen extend vertically so that the higher uncertainty in latitude was less likely to affect the extracted values.

## Results

From tags deployed in 2002–2005 on Guadalupe Island white sharks, four PAT tags were recovered after deployment periods of 5.4 to 8.2 months (Table 1). Three of the tags were physically removed from the sharks while at Guadalupe Island and one was found on Laysan Island in the northwestern Hawaiian Islands. The four tags were deployed on two different males and one female (for ease of interpretation, tag numbers are followed by the letters F or M to designate sex). Two of the tags were deployed on the same male in consecutive years (30M and 40M, Table 1). Tag 18M was deployed on a shark that was estimated at 4.9 m total length (TL), the shark carrying tags 30M and 40M was estimated at 3.4 m TL, and for the female shark (41F) the length was unknown.

**Table 1:** Tagging data for recovered white shark pop-up satellite tags deployed at Guadalupe Island Mexico.

Tag #/sex	Tagging date	Photo-ID number^a^	Total length	Pop-up date	DAL^b^	Date left Guadalupe Island	Date returned
18M	12/05/02	19	4.9	8/08/03	246	2/02/03	7/28/03
30M	12/05/03	10	3.4	8/06/04	245	5/05/04	7/30/04
40M	12/12/04	10	3.4	8/08/05	239	4/07/05	7/15/05
41F	12/07/05	64	?	5/15/06	162	2/10/06	

a Photo-ID numbers are from Domeier & Nasby-Lucas [17].

b DAL indicates the number of days that the tag was on the shark.

**Figure 1:**
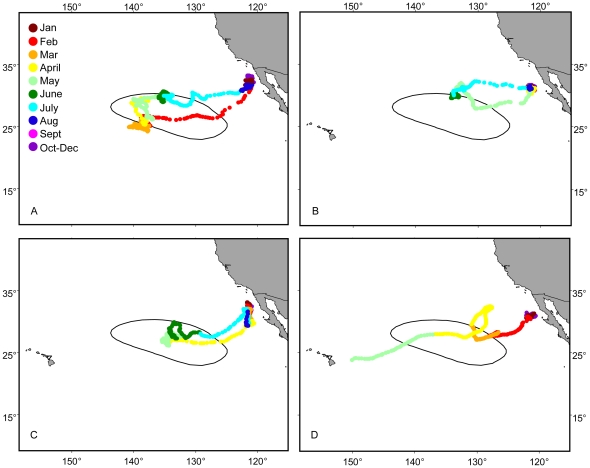
Offshore movement patterns for tagged white sharks. Calculated tracks for (A) tag 18M, (B) tag 30M, (C) tag 40M, and (D) tag 41F. The black line indicates the 50% fixed kernel contour for previous pop-up satellite archival tag pop-up points in the SOFA (Domeier & Nasby-Lucas 2008). Colors indicate months.

All four tags were deployed in the month of December, and the sharks began their seasonal migration away from Guadalupe Island between February and May (Table 1). Three tracks (tags 18M, 30M and 40M) showed offshore movement to the SOFA and the fourth track (41F) demonstrated a migration that moved through the SOFA and to the vicinity of Hawaii (Fig. 1). The SOFA [15] is an expansive pelagic habitat encompassing a large area between Guadalupe Island and the Hawaiian Islands between approximately 32° and 16°N latitude and approximately 128° and 142°W. The sharks that traveled to the SOFA remained there for 2.2 to 4.8 months and then returned to Guadalupe Island where the tags were recovered in early August. Movements to and from the SOFA were relatively direct and took between 10 and 20 days. Tag 41F moved offshore, spending two months swimming in a loop around the northeastern edge of the SOFA and then continued westward toward the Hawaiian Islands where the tag popped up 338 km east of the island of Hawaii in mid-May (Fig. 1). The female maintained directed movement during the entire offshore time period, in comparison to the males, which were still in the SOFA in May.

### Vertical Movements

Vertical movement patterns and vertical activity levels varied with region and between individuals with different migratory patterns. All sharks exhibited significant time at the surface (0–5 m) while migrating offshore away from Guadalupe Island (tag 18M 67.5%, 30M 71.5%, 40M 85.4%, and 41F 44.1%). While the female maintained this pattern of considerable time at the surface throughout the remainder of the track, the behavior of the male sharks changed dramatically when they arrived in the SOFA.

**Figure 2:**
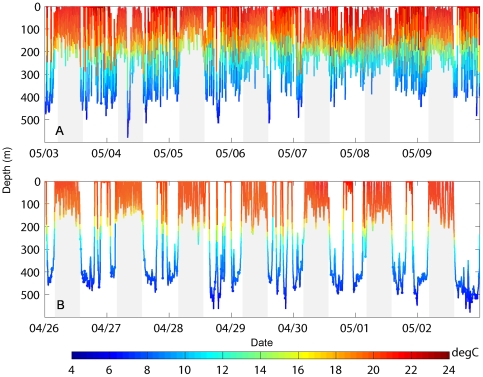
Characteristic offshore vertical profiles indicating dive behaviors in the SOFA and while traveling. Ambient temperature-depth profiles for one week with typical vertical behavior for (A) male 40M while in the SOFA and (B) female 41F while traveling to Hawaii. Color denotes ambient temperature. Light grey denotes nighttime.

The vertical movements of the male sharks in the SOFA consisted primarily of continuous oscillatory dives with quick repetitive descents and ascents with little time spent at depth before the shark initiated the next ascent or descent (Fig. 2a). It was not uncommon for these repetitive dives to extend for hundreds of meters. At night, the extensive vertical movements continued, although depths were shallower and near the boundary of the mixed layer (Fig. 2a). Throughout the course of the track, the males showed some variability in the duration of the daytime dives and amount of time at the surface. Longer surface-based dives were noted on 3 May with increased subsurface oscillatory movement on 8 May (Fig. 2a).

The vertical behavior pattern for 41F, the shark that traveled towards Hawaii, differed from the patterns exhibited by the male sharks. This shark typically made deep dives with an extended duration at a target depth during the day and shallow surface based dives within the mixed layer at night (Fig. 2b). Some variability in this pattern was apparent over the course of the track. The maximum dive duration for the female shark was 15.7 hrs. Although this length of dive was uncommon, during the entire period in the offshore region, 54.2% of the days for this shark had a daytime dive with duration greater than five hours. During the loop towards the north which lasted from late February to mid April (Fig. 1), this shark spent a mean percent time at the surface of 52.2±21.0% including 14.5% of days with a mean percent time at the surface of 75.1±15.1%. In comparison, when 41F moved towards the west, the mean time at the surface was less (29.8±18.5%). The bimodal depth pattern was observed during both periods despite the differences in surface time. This shark did not display the repetitive oscillatory diving pattern characteristic of the male sharks.

**Figure 3:**
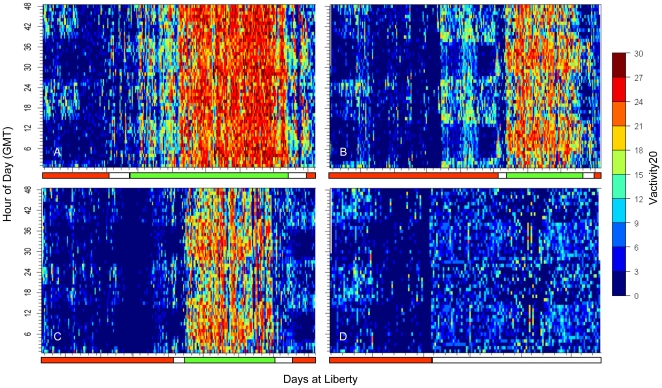
Vertical activity analysis for the tagged white sharks. V_activity20_ values for each of the four datasets displayed over 48 hours for the entire tracking period for tags (A) tag18M, (B) tag 30M, (C) tag 40M, and (D) tag 41F. The colored bar at the bottom indicates red for time at Guadalupe Island, white for traveling and green for time in the SOFA.

The difference in vertical behavior was highlighted by vertical activity analysis (V_activity20_), which indicated whether individual sharks were making large repetitive vertical movements (Fig. 3). The (V_activity20_) showed low average activity levels for all four sharks while at Guadalupe Island, with a range of from 2.3 to 4.6 (Fig. 3 and Table 2). Higher average levels of V_activity20_ occurred offshore for tags 18M, 30M and 40M with a range of 17.2 to 19.6, but not for tag 41F, which had an average V_activity20_ of 4.7 (Fig. 3 and Table 2). The Dunn's method for pair-wise multiple comparison indicated that there was a significant difference between all tag pairs but not between 30M and 40M, which were from the same shark. Although the average levels of V_activity20_ for 41F were low offshore, there were two days when the shark was near the northern edge of the loop where nighttime average V_activity20_ values were 15.5 and 13.3. For shark 18M, which spent the most time offshore, V_activity20_ changed over the course of the track (Fig. 3a). During the first six weeks within the SOFA, this shark stayed in a concentrated area towards the southwest (Fig. 1a) and spent 44.4±20.2% of time at the surface with a mean V_activity20_ of 11.7+7.5. During the next 12 weeks in the northeast portion of the SOFA, the shark showed a stronger oscillatory pattern; less time at the surface (10.6±12.5%), and a higher mean V_activity20_ (23.5±4.8) in a concentrated area on the northern edge of the track (Fig. 1).

**Table 2:** V_activity20_ values for tagged Guadalupe Island white sharks.

Tag #/sex	Ave V_activity20_ at Guadalupe Island	Ave V_activity20_ offshore	Ave offshore day V_activity20_	Ave offshore night V_activity20_
18M	3.5±4.5	19.6±8.0	19.5±8.4	19.8±7.3
30M	4.6±5.3	17.8±7.3	16.1±7.7	20.1±5.9
40M	2.3±3.4	17.2±7.6	15.6±8.0	19.5±6.5
41F	2.8±4.0	4.7±4.7	3.9±4.7	5.7± 4.6

A comparison of V_activity20_ between day and night showed that offshore 30M, 40M and 41F had increased activity at night (Fig. 3 and table 2). The V_activity20_ daytime average for 30M and 40M ranged from 15.6 to16.1, while nighttime was 19.5 to 20.1. The V_activity20_ daytime average 41F was 3.9, while nighttime was 5.7 (Table 2)(Mann-Whitney Rank Sum Test for all three tags, *p*<0.001). Unlike the other tags, 18M had similar average daytime and nighttime V_activity20_ values (19.5 and 19.8 [Mann-Whitney Rank Sum Test, *p* = 0.496]) (Table 2).

**Figure 4:**
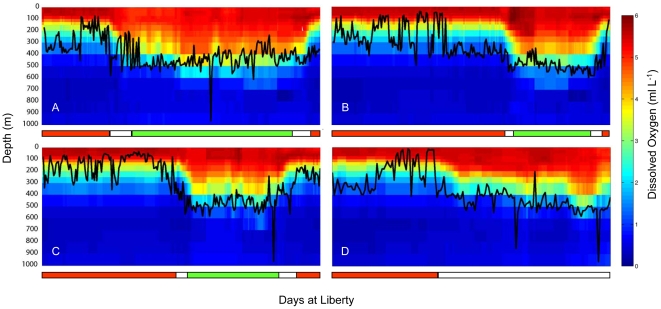
Oxygen and maximum depth profile for tagged white sharks. Maximum daily diving depths for the entire tracking period for tags (A) 18M, (B) 30M, (C) 40M and (D) 41F displayed over the WOA05 monthly mean O_2_ concentration extracted for each track point. The colored bar at the bottom indicates red for time at Guadalupe Island, white for traveling and green for time in the SOFA.

While vertical activity differed across the four tag datasets, the average and maximum daily diving depth values were similar while offshore. The maximum depth achieved was >979.5 m (exact depth is unknown since Wildlife Computer's tags do not measure depths greater than 979.5 m). Although all sharks made at least one dive deeper than 900 m (Table 3), 99.7% of all data from all four tags in the offshore region was above 500 m (Fig. 4). Average daily maximum depth values while in the offshore region ranged from 442.5 to 492.8 m (Table 3). The daily maximum depth values for 30M, 40M and 41F were not significantly different (Kruskal-Wallis one way analysis of variance on ranks p =  <0.06). Values for these three tag datasets were significantly different than for 18M (Kruskal-Wallis one way analysis of variance on ranks p =  <0.01), although the difference in depths is small.

**Table 3:** Temperature, depth and dissolved oxygen levels experienced by the tagged Guadalupe Island white sharks while offshore.

Tag #/sex	Max SST (°C)	Min SST (°C)	Min dive temp (°C)	Max depth (m)	Day average max depth (m)	Night average max depth (m)	Ave daily min O_2_ (ml L^−1^)	Min O_2_ (ml L^−1^)
18M	23.0	19.2	4.5	964	442.4±83.5	277.9±54.7	2.9±1.2	0.4
30M	22.3	19.1	5.4	596	492.8±50.6	291.2±55.0	2.1±1.0	0.4
40M	23.1	20.6	4.1	>979.5	469.0±47.6	332.3±86.1	1.8±0.7	0.3
41F	24.8	15.1	3.9	>979.5	472.2±95.9	245.3±116.3	1.7±0.8	0.5

**Figure 5:**
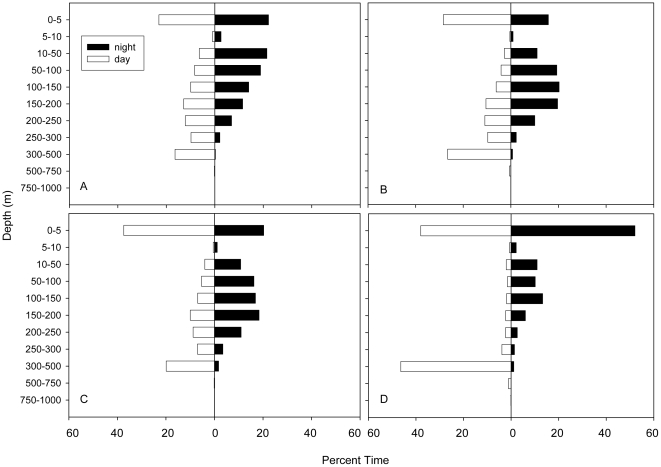
Day and night depth distributions for tagged white sharks while offshore. Plot of percent time of day and night depth distribution for tags (A) 18M, (B) 30M, (C) 40M, (D) 41F.

All sharks showed a strong diel pattern in vertical distribution while offshore. The daily average daytime and nighttime maximum depth values were significantly different for all tracks, with daytime values ranging from 442.4 to 492.8 m and nighttime from 245.3 to 332.3 m (Table 3, for all four tags Mann-Whitney rank sum test p = 0.001). The average depth of the mixed layer in the SOFA was approximately 120 to 132 m [14], [15]. Although all sharks showed diel differences in vertical distribution, this varied between the sharks that remained in the SOFA and the shark that moved towards the Hawaiian Islands (Fig. 5). During the day all sharks had time at the surface (0–5 m) of between 23 and 38%, while times below 300 m for the male sharks were between 16.5 and 27.1% and for tag 41F it was 47.6%. During the night, shark 41F spent 52.1% of the time at the surface while tags 18M, 30M and 40M spent between 15 and 22% there.

Rates of ascent and descent were examined for all tagged sharks. Tags 18M, 30M and 40M had mean rates of descent that were significantly faster than the mean rates of ascent while offshore (18M descent 0.9±0.2 m sec^−1^, ascent 0.6±0.1 m sec^−1^; 30M descent 0.9±0.2 m sec^−1^, ascent 0.6±0.1 m sec^−1^; 40M descent 1.0±0.2 m sec^−1^, ascent 0.6±0.1 m sec^−1^, for all three sharks, Mann-Whitney rank sum test p = 0.001). Shark 41F, which exhibited very different behavior in the offshore region, had mean rates of ascent and descent that were not statistically different, and both tended to be slower than those observed for the males (descent 0.3±0.2 m sec^−1^, ascent 0.3±0.1 m sec^−1^, Mann-Whitney rank sum test p = 0.8).

Regression analysis of mean nighttime depth and lunar illumination revealed a significant increase in depth as lunar illumination increased for the three male sharks but not for the female (regression analysis, p<0.05). The variation explained by the regression equation ranged from R^2^ = 0.3 to 0.54. Mean nighttime depth during new and full moon periods increased by approximately 20 m for 18M, 26 m for 40M, and 40 m for 30M.

### Temperature

Sharks experienced a broad range of temperatures over their migratory route. Sea surface temperature (SST) for all four sharks while offshore ranged from 15.5 to 24.8°C (Table 3). Minimum dive temperature experienced for each shark ranged from 3.9 to 5.4°C (Table 3) and maximum difference between ambient temperature and SST were comparable for all sharks and between 17.1 to 18.9°C.

**Figure 6:**
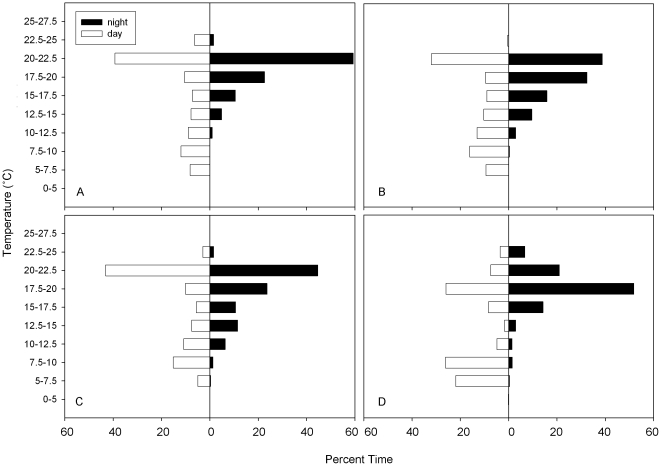
Day and night temperature distributions for tagged white sharks while offshore. Plot of percent time of day and night temperature distribution for tags (A) 18M, (B) 30M, (C) 40M, (D) 41F.

Temperature distributions by day and night indicated differences in diel patterns as well as differences between the sharks that remained in the SOFA compared to the shark that moved towards the Hawaiian Islands (Fig. 6). Daytime temperature distributions showed that while offshore shark 41F, which exhibited long dives to depth, spent more time during the day below 10°C (48.0%) than all male sharks. Male sharks spent between 20.1 and 25.5% of their time at temperatures below 10°C. Nighttime temperature distributions showed warmer temperatures with all sharks spending between 78.9% and 92.5% of the time between 15 and 22.5°C.

### Oxygen

**Figure 7:**
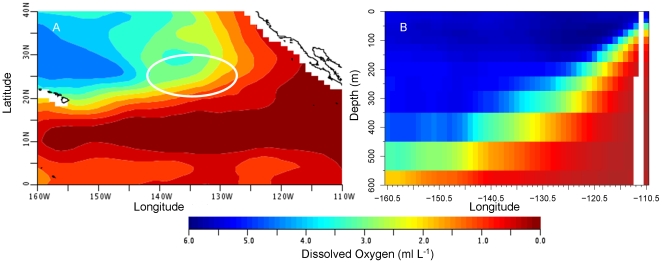
World Ocean Atlas 2005 dissolved oxygen levels in the eastern North Pacific. Mean O_2_ levels in the Eastern Pacific (A) at 400 m for the month of June with the white circle representing the approximate location of the SOFA, and (B) the profile of O_2_ at depth along 25.5° N latitude. Color indicates changes in mean O_2_ levels.

In moving from Guadalupe Island offshore, the depth of the oxygen minimum zone (defined as <1.5 ml L^−1^) increases (World Ocean Atlas [26]) (Fig. 7). Maximum daily depths were plotted with oxygen profiles derived from the World Ocean Atlas data (Fig. 4) and revealed that in general, the maximum daily depth values follow the contour of the oxygen minimum zone. At the maximum daily depth for all sharks, the average minimum dissolved oxygen ranged between 1.7 ml L^−1^ and 2.9 ml L^−1^ (Table 3). Minimum O_2_ levels likely to have been experienced for each shark ranged from 0.3 to 0.5 ml L^−1^ (Table 3) and were associated with relatively brief dives. Standard error values for the extracted data varied by location and depth with a range of 0.0 to 0.4 ml L^−1^ with the majority having an error less than 0.1 ml L^−1^.

**Figure 8:**
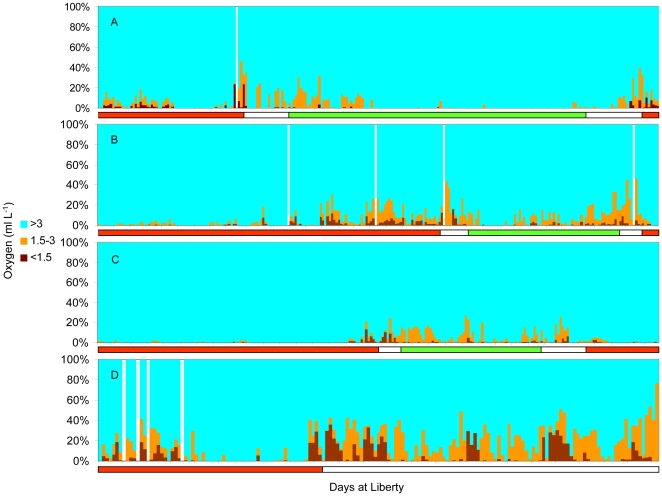
Percent time at dissolved oxygen levels for tagged white sharks. Histograms of percent time at dissolved oxygen levels for the entire tracking period for tags (A) tag 18M, (B) tag 30M, (C) tag 40M and (D) tag 41F. White lines indicate days with no data. The colored bars at the bottom indicate red for time at Guadalupe Island, white for traveling and green for time in the SOFA.

**Figure 9:**
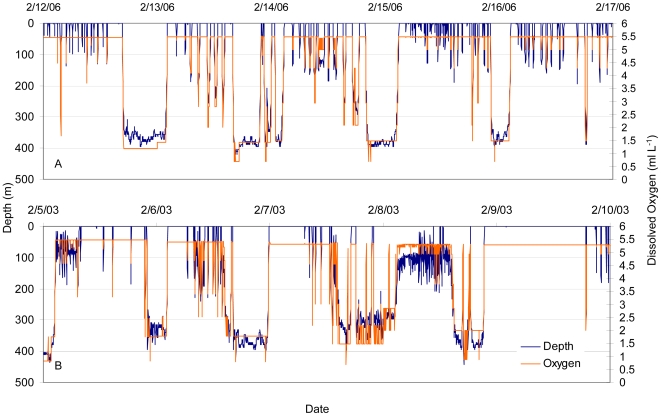
Depth and inferred oxygen profiles. Vertical profile showing estimated O_2_ levels experienced at depth for tag (A) 41F and (B) 18M shortly after leaving Guadalupe Island.

Looking at data binned by percent time spent at varying O_2_ concentrations indicated time spent in waters below 1.5 ml L^−1^ (Figure 8). For all four sharks the highest percent time at this level was experienced on the eastern edge of the Pacific, where the hypoxic zone is shallowest (Fig. 7). Maximum percent time of a 24-hour period below 1.5 ml L^−1^ dissolved oxygen was 37.5% for the female and between 13.5 and 23.6% for the males. For the female this occurred while beginning the offshore migration on 13 February 2006, and she was estimated to spend as much as 9.0 hours continuously during a single dive in waters with a dissolved oxygen concentration just below 1.5 ml L^−1^ (average O_2_ concentration over the 9 hrs was 1.2±0.1 and average depth 361.8±14.9 m) (Fig. 9a). For the males, periods with maximum percent time below 1.5 ml L^−1^ were not continuous. Tag 18M spent 23.6% of time just below 1.5 ml L^−1^ with 1.7 hours at an O_2_ concentration of 1.49 ml L^−1^ including one data point 0.7 ml L^−1^ (Fig. 9b).

### Extreme Dives

The greatest range in depth, temperature, and oxygen was experienced during the four previously mentioned dives that exceeded 850 m (Fig. 4). The deepest dive by the female shark (41F) lasted for 12.2 hours with an average depth of 524.9±134.6 m and average temperature of 7.4±1.9°C. This dive included 28 min at depths greater than 979.5 m where the temperature was as low as 3.9°C, without returning to the surface for another 7.6 hours. At the deepest part of this dive, the light readings plateaued for 124 min indicating the absence of any light, and the shark experienced 3 hours in hypoxic water with O_2_ levels that ranged from 0.5 to 1.2 ml L^−1^ (average 1.0±0.2 ml L^−1^). This dive occurred on 12 May 2006, six days before the end of the track between the SOFA and Hawaii. It began 0.3 hours before sunrise and ended 1.6 hours before sunset and was followed by 4.0 hrs at the surface.

The deep dives for the males were shorter with less time in hypoxic water. The deepest dive for shark 18M was 964 m (Fig. 4a). This dive lasted 4.5 hours with an average depth of 455.8±128.0 m and average temperature of 7.6±1.8°C. This shark experienced 12 minutes in hypoxic water with an O_2_ concentration of 0.6 ml L^−1^. This dive occurred on 3 May 2003 in the SOFA. The deepest dive for 40M was to depths greater than 979.5 m (Fig. 4c). This dive lasted 3.5 hours with an average depth of 463.6±197.3 m and average temperature of 7.8±3.9°C. This shark spent 28 minutes in hypoxic water, where minimum O_2_ level ranged from 0.3 to 0.8 ml L^−1^ (average 0.5±0.2 ml L^−1^). This dive occurred on 28 June 2005 at night while traveling back toward Guadalupe Island.

## Discussion

While the nearshore behavioral patterns of white sharks have received considerable attention and are relatively well understood, little has been known about their offshore habitats and habitat use [13]–[15]. This study represents the largest archival tag dataset analyzed to date to provide detailed information on the behaviors and habitat use of white sharks while offshore in the North Pacific Ocean. The detailed archival records provided the opportunity to examine behavioral differences that are challenging to infer from daily histograms. The archival datasets reveal that these sharks display diverse behaviors and can tolerate very cold temperatures and extremely low oxygen levels. This study provides a novel and more complete characterization of the offshore movements of white sharks in this area relative to environmental factors.

### Vertical Movement Patterns

An examination of vertical movements reveals two distinct patterns that appear to be related to phases of residency in the offshore region or migration. The first is characterized by an oscillatory dive behavior and the second by a bimodal depth distribution with substantial surface time. The ability to use vertical movements to differentiate between possible foraging and migratory behaviors will dramatically enhance efforts to use state-space models to quantify behavioral modes [27]–[29].

All sharks exhibited significant time at the surface while traveling to the SOFA, consistent with migratory behavior. Once in the SOFA, the male sharks switched behavioral modes and exhibited constant repetitive oscillatory dive patterns. While the oscillatory dive pattern was consistent across tracks, the intensity in vertical activity varied between the tracks for the two male sharks with varying patterns in different areas of the SOFA for 18M. The vertical activity patterns for the two tags from the same shark (30M and 40M) were very similar for both years of tracking, suggesting that individuals may have preferred and consistent behaviors across years.

Unlike the males that remained in the SOFA, the female maintained relatively directed movement and did not reside in any one area for a protracted period. Through the majority of the tracking period, she made longer dives with more time at depth during the day. She also exhibited a bimodal depth distribution split between time spent at the surface and at greater than 300 m. This pattern is similar to that of other tracked female white sharks traveling from central California to south of the Hawaiian Islands [14] and from South Africa to Australia [18]. While these sharks were all females, males have also been tracked to Hawaii and females have been shown to remain in the SOFA [14], [15], so the discrepancy in behavior is likely not sex-dependent. A larger sample size would allow a better comparison of the differences in migration patterns and vertical diving behavior between the sexes.

It has been documented in this and other studies that considerable time at the surface, punctuated with deep dives, is a typical traveling behavior pattern [13]–[15]. Surface swimming may be related to geomagnetic navigation. One theory is based on light-dependent magnetic orientation, where light absorption by photosensitive molecules initiates magnetosensitive chemical reactions and allows for magnetorecption [30], [31]. A related hypothesis states that the earth's main dipole field is most uniform at the surface but the magnetic gradients are steeper and more perceptible at depth, thus both surface swimming and deep dives are necessary [32]. Although it is not clear exactly what is dictating the behavior, it does appear that navigation in white sharks relies on extended surface swimming. The fact that our female shark (41F) maintained this bimodal vertical behavior pattern throughout the entire track suggests that the tag came off prior to the shark reaching her destination. The shark tracked for 194 days by Weng at al. [14] to Hawaii did shift her vertical behavior pattern once she reached the Hawaiian Islands.

All sharks displayed vertical movements both day and night, but there were diel differences, with deeper depths during the day than at night. The sharks that traveled to the SOFA continued their oscillatory dives at night but remained primarily in the mixed layer. A similar diel shift in depth distribution has been observed in a range of pelagic fish including swordfish (*Xiphias gladius*) [33], bigeye thresher shark (*Alopias superciliosus*) [34], bigeye tuna (*Thunnus obesus*) [35], megamouth shark (*Megachasma pelagios*) [36] the school shark (*Galeorhinus galeus*) [37], and jumbo squid (*Dosidicus gigas*) [38]. Diel patterns in vertical movements are generally thought to be indicative of foraging on species associated with the vertically migrating DSL [33], [39], [40].

The vertical movement patterns of the white shark suggest an association with the DSL both during the day and night. The depth of the DSL is light-dependent and is thus influenced by both lunar and solar illumination [41]–[43]. The estimated depth at the top of the DSL in this region during the day was 460 m and quite similar to the average daily maximum depths for these tagged sharks. The top of the DSL was estimated using the equation provided by Tont [42] for the Pacific, which utilizes a conversion between Secchi depth and the attenuation coefficient [44] and the regional satellite-derived attenuation coefficient at 490 nm (kd 490). Improved information on the depth and composition of the DSL would help to better define the relationship between the DSL and the maximum diving depths, but this analysis shows that the maximum diving depths are in the general area of the DSL. The average nighttime depths for these sharks while in the SOFA increased during the full moon, which further supports the hypothesis that they are associated with the DSL. The female shark, on the other hand, showed no shift in nighttime depth with changing illumination. While she remained in the mixed layer at night, overlapping with the DSL, it isn't clear if she was foraging.

Assuming the sharks that remain in the SOFA are in fact foraging as has been suggested in this and other studies [14], [15], it is probable that the oscillatory dive pattern is associated with foraging. While oscillatory hunting strategies have been observed across a range of species, they are not well understood. The purpose of the oscillatory diving behavior may be to move between two strata of water to track chemical cues [32] or perhaps utilize visual cues which are enhanced when looking up against down-welling light [39]. Certainly in the nearshore environment white sharks typically attack from below [21], [45]. It is not likely that the pattern observed is associated with the burst-glide swimming mode observed in many pelagic animals [46], [47] to increase efficiency. The rates of ascent are less than of descent and are the reverse of what was proposed by Weiss [48].

While the SOFA is not well characterized oceanographically or biologically, some insights into potential prey come from information on fisheries in the area. Potential fish prey species based primarily on longline catch data include albacore (*Thunnus alalunga*), yellowfin (*Thunnus albacares*) and bigeye tuna, as well as swordfish, with other billfishes and sharks present in smaller numbers [49], [50]. Cetaceans, if present, would be other potential prey species. Future trips to the SOFA or biochemical analysis of tissue composition may provide evidence of what these sharks are preying on and what environmental cues they may be following.

### Temperature and Oxygen

In an effort to better understand behaviors it is informative to examine the potential limitations to movements imposed by physiological constraints associated with varying environmental conditions. Understanding physiological limitations can help differentiate between shifts in distribution associated with some resource, such as prey, or the influence of a physiological stressor. The predominant environmental parameter commonly reported to influence movements and behaviors is temperature, while oxygen has received more attention recently. The impact of oxygen is typically in the vertical plane, especially in regions like the eastern Pacific where the oxygen minimum zone is close to the surface [33], [49], [51]. Both temperature and oxygen show strong vertical clines along the tracks of white sharks.

To gain insight into white shark physiology we can look to comparative studies of other lamnid sharks and tunas when data for white sharks are not available. While there is variation within both groups, considerable convergent evolution has been demonstrated. Both groups possess a suite of similar adaptations that enhance performance, from the structure of the heart to the evolution of endothermy (summarized in Bernal et al. [52]). Clearly, additional opportunistic research is needed on white shark molecular biology, biochemistry, physiology, and the impact of ambient temperature and oxygen concentration.

As mentioned, temperature influences the vertical and geographic distributions of pelagic fishes, and avoidance of water temperatures too warm or two cold have both been reported [47], [53]–[55]. As temperature increases or decreases beyond an optimal range, cardiac, neural and muscular function can be compromised. For white sharks a loss of function in cold waters is minimized (and potentially negated) due to their endothermic ability and their large body size [56], [57]. In fact, data indicate that white sharks defend a core body temperature of approximately 26°C in waters down to around 12°C [10], although no data are available offshore where considerably colder temperatures are experienced. Their impressive thermal range associated with vertical movement is however not surprising given their large latitudinal range. White sharks have been documented as far north as the Bering Sea [58]. An examination of the two behavioral modes gives no indication that dives are driven by behavioral thermoregulation. The frequent repetitive vertical movement dives are not characteristic of behavioral thermoregulation, where there tends to be a plateau at depth, presumably associated with foraging, and sometimes another at the surface for body temperature to recover [39], [59], [60]. Further evidence that the behavior is not thermoregulatory is the fact that the female was capable of spending long periods in cold water. The deepest dive for this shark was to greater than 1000 m, with an ambient temperature of 3.9°C, without returning to the surface or mixed layer for another 7.5 hours.

While it is likely that body-core temperature remains substantially elevated above water temperature during the dives, the question that remains is how the sharks are able to maintain cardiac function and support aerobic metabolism at low temperatures. Given the long dives of the female in particular, metabolism is most likely supported by aerobic processes. The heart is not served by counter-current heat exchangers and operates at ambient water temperature [54], [61], [62]. To maintain cardiac function at low temperatures, it is possible that white sharks possess adaptations similar to those found in the closely related salmon sharks (*Lamna ditropis*). Salmon sharks, with a more boreal distribution than white sharks [54], [63], show an even more extreme tolerance for cold waters [64]. The salmon shark's ability to maintain cardiac function in cold water has been linked to the up-regulation of proteins required for excitation-contraction coupling and consequently, maintaining heart rate [54]. These coupling proteins are up-regulated by an order of magnitude in comparison to blue sharks. Although not quantified, white sharks also show relatively high concentrations of these coupling proteins [54]. Increased expression of the same proteins has been shown in hibernating mammals that are resistant to cardiac dysfunction at cold temperatures [65].

The precise oxygen concentration that marks hypoxia in white sharks could not be determined in this study, but it is possible to estimate approximate concentrations. The maximum daily dive depth corresponded to climatological dissolved oxygen concentrations between 1.7 and 2.9 ml L^−1^. Looking at individual dives in figure 9, the sharks only made brief excursions much below 1.5 ml L^−1^. Given these observations and the errors associated with using the World Ocean Atlas climatology data, the lower limit is likely between 1.5 and 2.0 ml L^−1^. This limitation appears to constrain vertical movements to the top 500 m while traveling from Guadalupe Island. Bonfil et al. [18] reported a percent time of 18% at depths greater than 500 m while offshore in the Indian Ocean (where O_2_ levels at 500 m are approximately 4.8 ml L^−1^) in comparison to 0.7% in this study for 41F. Unfortunately, little information is available on hypoxia tolerance in sharks, and none is available for endothermic sharks. In comparison to other species, the levels reported are lower than those for yellowfin and skipjack (*Katsuwonus pelamis*) but comparable to those for bigeye tuna and swordfish [33], [49], [66].

The ability to tolerate low oxygen levels is largely dictated by the oxygen binding characteristics of the blood. Given that physiological data are not available for active pelagic sharks, the best available comparison is to bigeye tuna [66]. Bigeye tuna are also active pelagic fish, are endothermic, experience relatively low water temperature and most importantly have been documented in waters to ∼1 ml L^−1^ O_2_ [49]. An analysis of bigeye tuna blood binding characteristics by Lowe et al. [66] indicated a high oxygen binding affinity at the gills and a large decrease in affinity as the blood is warmed while passing through the counter current heat exchangers. Both these features enhance oxygen delivery in low oxygen environments. Bigeye tuna forage on organisms associated with the DSL which extend into the oxygen minimum zone in the eastern Pacific [49]. While it is not currently possible to determine if white sharks possess similar adaptations to those of bigeye tuna, this study reveals that it is possible for an active endothermic fish to function aerobically in waters with O_2_ concentrations at 1.5 ml L^−1^.

While little is known about blood binding characteristics, it is known that lamnid sharks possess adaptations that enhance oxygen transfer across the gills, increase the quantity of oxygen transported by the blood, elevate the delivery rate of oxygenated blood to tissues, and facilitate the intracellular transport of oxygen to the mitochondria. Lamind sharks have a larger gill surface area than that of most other fishes, which enhances oxygen uptake at the gills [52]. They also have a shorter blood-water diffusion distance, a complex capillary-muscle fiber geometry that significantly increases the ratio of capillary surface area to muscle fiber volume in the red muscle [67], and high levels of myoglobin [68]. While the vast majority of time was spent at O_2_ levels above 1.5 ml L^−1^, sharks in this study were still capable of making excursions to depths with O_2_ levels of approximately 0.5 ml L^−1^. Dives such as the one 48-min dive to below 1 ml L^−1^ are likely to be partially supported by anaerobic metabolism.

While additional information on specializations for low temperature and oxygen levels are needed to better define constraints to vertical movements, it is evident that white sharks possess a suite of cardiovascular and hematological characteristics which accompany endothermy. These characteristics allow utilization of a larger part of the ocean environment. This affords the opportunity to occupy waters out of the range of other species and reach the depth of the DSL in the SOFA.

### Conclusions

While we are beginning to characterize the behaviors and habitat preferences of white sharks in the SOFA, the question still remains as to what draws white sharks to this area. Our vertical-behavior analyses provide evidence suggesting that while in the SOFA the sharks are following the DSL and potential prey animals that are feeding within/upon the DSL. White sharks possess unique physiological traits that give them a predatory advantage when they exploit deep vertical habitats with gradients of structure (light, temperature, pressure, dissolved oxygen). While this is somewhat speculative, we hypothesize that the key to what draws these sharks to this region may lie in the vertical habitat and the habitat availability of potential prey species. The SOFA is situated against the edge of the oxygen minimum zone, which may act to compress prey distributions for the white sharks both vertically and horizontally. The low oxygen at depth may create a floor for the vertical movements. From a horizontal perspective, because the daytime depth of the DSL is not impacted by hypoxia in this region [42], the organisms that typically forage on the DSL won't be able to access its prey in regions to the south and east with a shallower oxygen minimum zone [49]. Essentially, the steep gradient may act to compress prey horizontally against the slanted wall of hypoxic water. Future studies using real-time tracking data may allow us to analyze precise geolocation data with environmental data to assist in directly observing the vertical structure of the water column as well as quantifying the ecology of the SOFA, perhaps more precisely identifying potential prey species.
